# When Time Stands Still: Age Differences in Temporal Disintegration During the COVID-19 Pandemic

**DOI:** 10.1093/geroni/igaf122.2312

**Published:** 2025-12-31

**Authors:** Teagan McCune, Meghan Elliot, Kayley Estes, Alison Holman, Roxane Silver

**Affiliations:** University of California, Davis, Los Gatos, California, United States; University of California, Irvine, Irvine, California, United States; University of California, Irvine, Irvine, California, United States; University of California, Irvine, Irvine, California, United States; University of California, Irvine, Irvine, California, United States

## Abstract

The COVID-19 pandemic was globally disruptive and life-altering. During collective traumas like the pandemic, some individuals experience temporal disintegration (TD), defined as a distorted sense of the passage of time (e.g., speeding up, slowing down, stopping). Numerous studies have shown that TD is associated with psychological distress following trauma exposure. While research on age differences in TD is limited, some studies suggest older people may experience less TD. The current study explored self-reported TD over the first two years of the pandemic among a nationally representative U.S. sample of adults (ages 18-97) who completed two online surveys (Sept-Oct 2020, N = 5,654; Nov 2021, N = 4,856). Weighted linear regressions assessed associations between TD, negative life events, emotional distress, and age groups. Both middle-aged and older adults experienced less TD compared to younger counterparts. Higher TD was also associated with having previously experienced more negative life events. While higher TD was associated with greater emotional distress, the relationship was significantly weaker in older adults compared to young adults; middle-aged adults did not show this weakening relationship with age. Results suggest that older adults may experience less distress in response to distorted time perceptions, perhaps because they have age-related positivity biases, suggesting greater adaptation to disruptions in time perception. These age-group differences may also be a result of increased self-continuity and time stability with age, a topic that future research should further explore. Future studies should also explore potential mechanisms underlying the age differences in TD.

